# Development and validation of a combined ultrasound−radiomics model for assessing rheumatoid arthritis disease activity: a prospective, two−center diagnostic study

**DOI:** 10.3389/fimmu.2026.1669766

**Published:** 2026-04-27

**Authors:** Jinjin Peng, Wenjing Guo, Jundong Yao, Haixia Lv, Biao Wang, Xinfeng Wu, Shuai Cui, Shengjiang Chen, Zhoulong Zhang

**Affiliations:** 1 Department of Ultrasound, The First Affiliated Hospital, College of Clinical Medicine of Henan University of Science and Technology, Luoyang, Henan, China; 2 Department of Ultrasound, The First Affiliated Hospital of Henan Medical University, Xinxiang, Henan, China; 3 Department of Ultrasound, Affiliated Hospital of Traditional Chinese Medicine, Xinjiang Medical University (Fourth Clinical Medical College, Xinjiang Medical University), Urumqi, Xinjiang, China; 4 Department of Rheumatology and Immunology, The First Affiliated Hospital, College of Clinical Medicine of Henan University of Science and Technology, Luoyang, Henan, China

**Keywords:** artificial intelligence, disease progression, machine learning, rheumatic diseases, rheumatoid arthritis, ultrasonography

## Abstract

**Objective:**

To develop and evaluate a combined model integrating musculoskeletal ultrasound (MSK US) with a machine learning (ML) algorithm for assessing disease activity in rheumatoid arthritis (RA).

**Methods:**

A total of 203 patients with clinically confirmed RA were prospectively enrolled from December 2023 to September 2025. A cohort of 142 patients from the First Affiliated Hospital, College of Clinical Medicine of Henan University of Science and Technology served as the training cohort, while 61 patients from Affiliated Hospital of Traditional Chinese Medicine, Xinjiang Medical University (Fourth Clinical Medical College, Xinjiang Medical University) constituted the independent external test cohort. Three predictive models were developed: (1) an MSK US model incorporating two-dimensional grayscale ultrasound, power Doppler ultrasound (PDUS), and superb micro-vascular imaging (SMI); (2) a radiomics model based on two-dimensional grayscale images using the extremely randomized trees (ExtraTrees) algorithm; and (3) a combined model integrating the first two. Model performance in assessing RA disease activity was evaluated and compared using receiver operating characteristic (ROC) curve analysis. Calibration curves and decision curve analysis (DCA) were subsequently used to validate the overall performance of the optimal model.

**Results:**

Multivariate logistic regression analysis identified erythrocyte sedimentation rate (ESR)>54 mm/h, C-reactive protein (CRP)>32.83 mg/L, and SMI synovial blood flow grade III as independent predictors of clinically active RA. The area under the ROC curve (AUC) values for the MSK US model, radiomics model, and combined model were 0.935 (95% confidence interval [CI]: 0.893-0.978), 0.976 (95% CI: 0.955-0.997), and 0.998 (95% CI: 0.998-1.000), respectively, in the training cohort; in the independent external test cohort, the AUC values for the three models were 0.904 (95% CI: 0.825-0.983), 0.823 (95%CI:0.714-0.933), and 0.929 (95%CI:0.866-0.992),respectively. The discriminative performance of the combined model was significantly superior to that of either the MSK US model or the radiomics model alone. Calibration curves demonstrated good agreement between the observed risk levels and the predicted risk probabilities. Decision curve analysis indicated that the model provided significant net benefit across threshold probability ranges of 0.02–0.80 in the training cohort and 0.12–0.78 in the test cohort.

**Conclusion:**

The combined model developed based on MSK US and radiomics demonstrated satisfactory performance for assessing disease activity in RA, enabling clinicians to dynamically monitor RA disease activity and evaluate treatment response, thereby providing a reliable imaging basis for the selection of routine medical treatment strategies.

## 1 Introduction

Rheumatoid arthritis (RA) is an autoimmune disease characterized by chronic synovitis and progressive joint destruction, frequently resulting in substantial joint dysfunction and reduced quality of life (1, 2). Current RA management aims to achieve and maintain clinical remission or low disease activity—a goal that hinges on accurate, dynamic assessment of disease activity (3).

Imaging therefore plays a critical role in RA management. Musculoskeletal ultrasound (MSK US), with its non−invasive, real−time, and high−resolution capabilities, allows earlier and more sensitive detection of joint effusion, synovial hyperplasia, blood−flow signals, and early bone erosion, establishing it as a key tool for early diagnosis and monitoring in RA (4, 5). The interpretation of ultrasound findings, however, must be grounded in histopathology (6). Rheumatoid arthritis synovitis is a dynamic process whose acute and chronic phases exhibit distinct pathological features that correspond directly to different sonographic appearances. The acute phase is characterized by exudation and acute inflammation. Histopathological hallmarks include synovial erosion, edema, and neutrophil infiltration, with capillary dilation as the predominant vascular change. Sonographically, this phase typically presents as joint−cavity effusion without marked synovial thickening; power Doppler often reveals no flow or only sparse punctate signals. In contrast, the chronic phase demonstrates marked synovial hyperplasia, fibrosis, and chronic inflammatory−cell infiltration. Grayscale ultrasound shows linear, villous, or pseudotumoral synovial thickening. Doppler ultrasound detects intra−synovial blood−flow signals reflecting inflammatory activity. Diffuse vascularization throughout the thickened synovium usually indicates high−activity chronic synovitis. Accurate quantification of these imaging features is therefore essential for staging inflammation and guiding individualized therapy.

Commonly used semiquantitative ultrasound scoring systems, however, are highly subjective, operator−dependent, and lack reproducibility and objectivity, limiting their ability to capture the continuous patho−radiological spectrum described above (7). Artificial intelligence (AI) has been applied in rheumatologic imaging along two main avenues to address this limitation. The first is deep−learning (DL)−based end−to−end image analysis; for instance, Fiorentino et al. used convolutional neural networks to automate selection of informative ultrasound frames (8). Such methods, however, generally demand large datasets and offer limited interpretability. The second avenue employs traditional machine−learning (ML)−based integrated predictive modeling. Illustrative work includes studies by Hu et al. (9) and Salehi et al. (10), which improved prediction of progression from undifferentiated arthritis to RA and estimation of time to clinical RA onset, respectively, by combining ultrasound scores with clinical features. These efforts underscore the ability of ML to integrate multi−source data while delivering interpretable predictions.

Despite these advances, existing research has focused largely on predicting RA onset. For the objective, fine−grained, imaging−based quantification of disease activity status in patients with established RA, current approaches remain inadequate. Neither the “black−box” character of DL nor the reliance of existing ML models on subjective, semi−quantitative ultrasound scores fully exploits the microscopic, continuous quantitative information present in raw images—information that often exceeds human visual perception. As a result, key imaging biomarkers reflecting disease−activity heterogeneity may be missed.

Radiomics, which extracts high−throughput quantitative features from medical images, offers a promising means to close this gap and has demonstrated considerable value in fields such as oncology (11–14). Its application to MSK US−based assessment of RA disease activity, however, is still in an early exploratory stage. Hence, this study aims to develop and validate a combined model that integrates conventional ultrasound metrics with radiomics features, using MSK US images and the extremely randomized trees (ExtraTrees) algorithm. We seek to build an assessment tool that is accurate, interpretable, and clinically applicable, thereby providing a novel method for the objective, precise quantification of RA disease activity.

## 2 Materials and methods

### 2.1 Study participants

**Figure 1:**
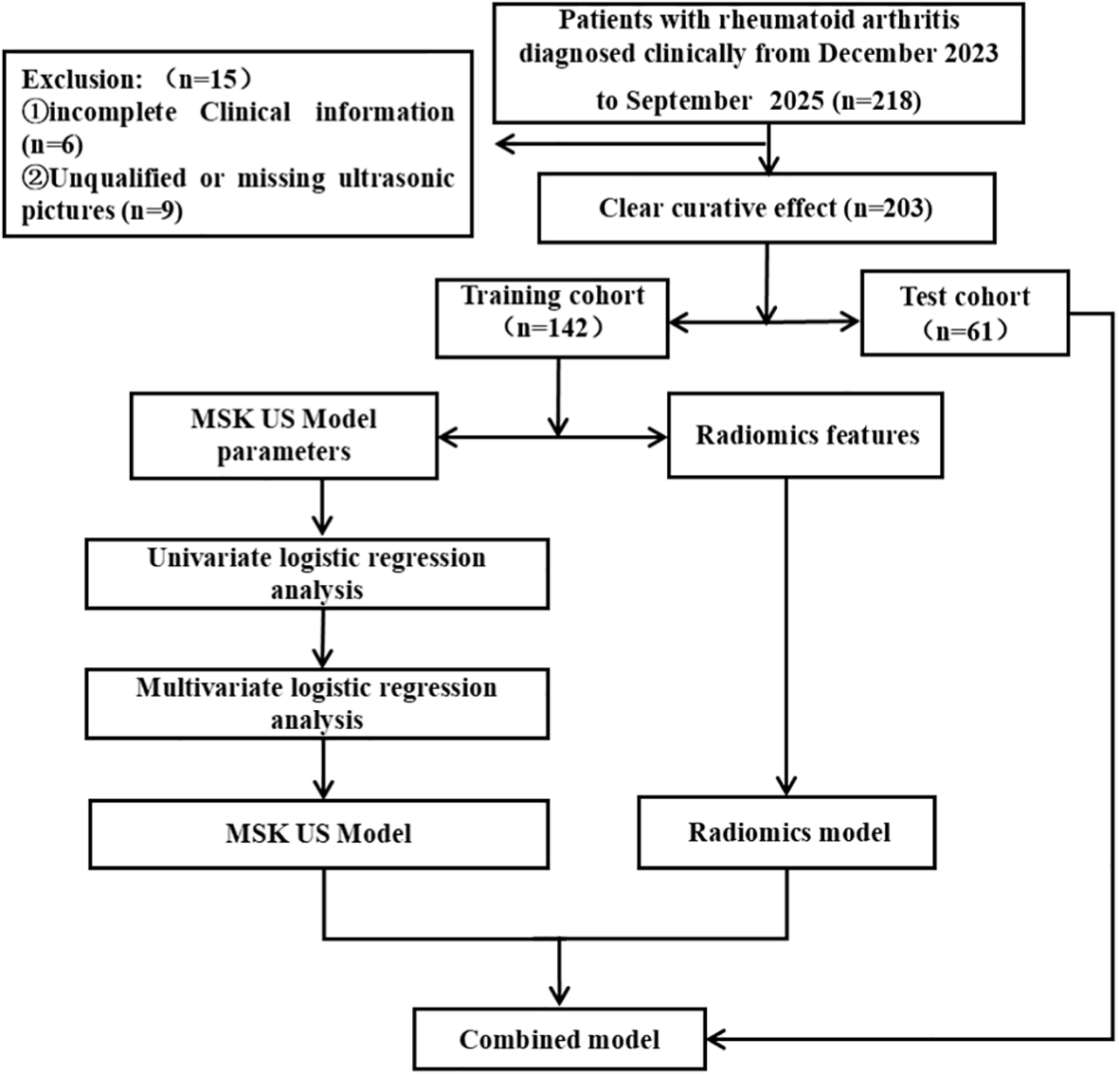
Flowchart of patient enrollment.

A total of 203 patients with wrist pain who were clinically diagnosed with RA were prospectively enrolled from December 2023 to September 2025. The inclusion criteria were as follows: (1) meeting the 2010 American College of Rheumatology/European League Against Rheumatism classification criteria for RA (15); (2) availability of complete clinical data and images. The exclusion criteria were as follows: (1) history of hand trauma; (2) presence of osteoarthritis, infectious arthritis, or metabolic arthritis; (3) inability to cooperate with the examination. Finally, 203 patients were enrolled, including 142 from the First Affiliated Hospital, College of Clinical Medicine of Henan University of Science and Technology and 61 from Affiliated Hospital of Traditional Chinese Medicine, Xinjiang Medical University (Fourth Clinical Medical College, Xinjiang Medical University). Patients were divided into cohorts based on study center: 142 patients from the First Affiliated Hospital, College of Clinical Medicine of Henan University of Science and Technology served as the training cohort, and 61 patients from Affiliated Hospital of Traditional Chinese Medicine, Xinjiang Medical University (Fourth Clinical Medical College, Xinjiang Medical University) served as the independent external test cohort. All patients underwent clinical disease activity assessment by a rheumatologist on the same day as the ultrasound examination. Using the Disease Activity Score 28−erythrocyte sedimentation rate (DAS28−ESR) score, patients were classified into clinical remission (DAS28 < 2.6) and clinically active (DAS28 ≥ 2.6) groups. This clinical assessment result served as the reference standard for this study and was used for all subsequent model development and validation. **Figure 1** shows the patient enrollment flowchart. The study protocol was approved by the Ethics Committee of the First Affiliated Hospital, College of Clinical Medicine of Henan University of Science and Technology (Approval No. 2024−03−K0158), and written informed consent was obtained from all participants.

### 2.2 MSK-US examination and image feature assessment

All examinations were performed using a Toshiba Aplio 500 ultrasound system with a 14−MHz high−frequency linear array probe. Patients were examined in a standard seated position with the symptomatic hand extended and resting flat. The built−in musculoskeletal preset was applied throughout the scan.

Each affected wrist was scanned in transverse and longitudinal planes from both palmar and dorsal aspects. Every standardized plane was assessed three times, and the mean value was recorded. To preserve low−velocity microvascular flow, the “ suspension technique “ was strictly followed: sufficient coupling gel was applied so the probe contacted the skin lightly without pressure. Depth was fixed at 2.5 cm. Focus and gain were optimized to obtain clear two−dimensional images. Joint effusion, synovial hyperplasia, and bone erosions were evaluated and semi−quantitatively graded using the Szkudlarek scoring system (16), defined as follows. Joint effusion grade: Grade 0, no effusion; Grade I, minimal effusion; Grade II, moderate effusion (without capsular distension); Grade III, large effusion (with capsular distension). Synovial hyperplasia grade: Grade 0, no synovial hyperplasia; Grade I, mild synovial hyperplasia, not extending above the line connecting the tops of the periarticular bones; Grade II, synovial hyperplasia extending above this line but not beyond the diaphysis; Grade III, synovial hyperplasia extending above the line and proliferating beyond the diaphysis on one side. Bone erosion grade: Grade 0, no bone erosion; Grade I, irregular bone surface without a definitive cortical break; Grade II, presence of a cortical break (bone defect); Grade III, bone defect causing extensive destructive changes (requires confirmation in two perpendicular planes).

Doppler settings were optimized according to established principles (17). Velocity scale, color gain, pulse repetition frequency, and sample−box size were adjusted to display synovial flow clearly while minimizing noise, ensuring diagnostically adequate image quality.

On the plane showing the most prominent synovial hyperplasia, the probe position was fixed. Without pressure, the mode was switched sequentially to power Doppler (PDUS) and then superb micro−vascular imaging (SMI) on the same plane to assess intra−synovial flow. Signals were graded semi−quantitatively (Szkudlarek system) and images were archived. Synovial blood flow grade: Grade 0, no blood−flow signal detected; Grade I, 1–2 punctate flow signals detected; Grade II, 3–4 short linear flow signals detected, involving ≤50% of the synovial area; Grade III, arborizing or network−like flow signals detected, involving >50% of the synovial area.

All scanning, parameter adjustments, and image acquisitions were conducted by two sonographers(Jinjin Peng, Wenjing Guo) who underwent unified training and adhered to the above protocol to ensure consistency. The standardized imaging procedure is illustrated in **Video 1**.

### 2.3 Construction of the MSK US model

MSK US parameters included bone erosion grade, joint effusion grade, synovial hyperplasia grade, PDUS synovial vascularity grade, and SMI synovial vascularity grade. Clinical characteristics included sex, age, ESR, rheumatoid factor (RF), and C-reactive protein (CRP).Stratified analyses were performed for age, ESR, RF, and CRP in this study. In the training cohort, univariate logistic regression analysis was conducted on MSK US parameters and clinical characteristics. Variables with *P* < 0.05 from the univariate analysis were selected for inclusion in the multivariate logistic regression analysis, using forward stepwise regression based on the likelihood ratio (LR) to identify independent predictors for building the MSK US model.

### 2.4 Construction of the radiomics model

**Figure 2:**
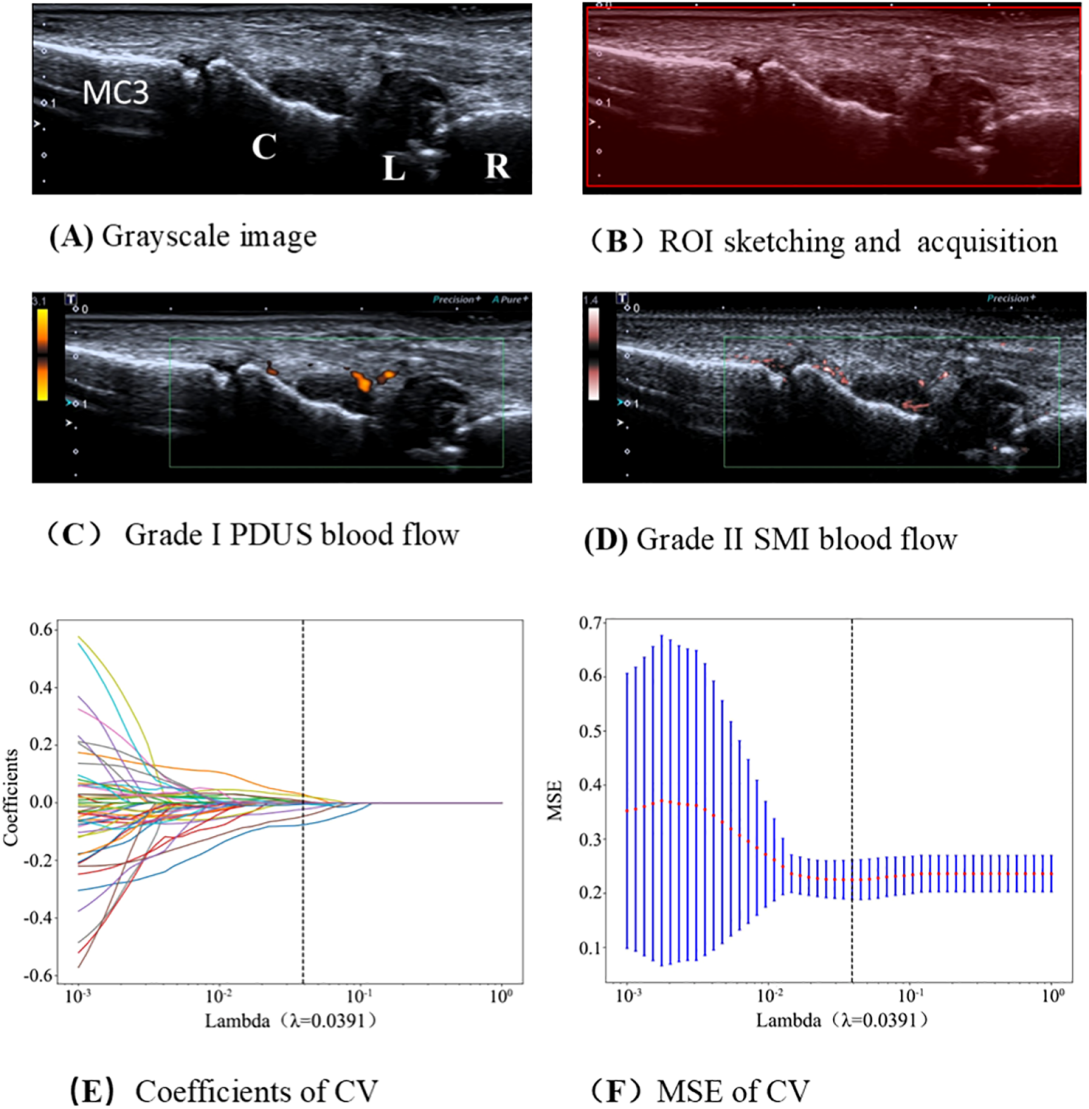
Flowchart of radiomics model construction. CV, cross−validation; MSE, mean squared error; PDUS, power Doppler ultrasound; SMI, superb microvascular imaging. R, radius; L, lunate; C, capitate; MC3, third metacarpal; ROI, region of interest.

All image selection and preprocessing were performed by a single trained sonographer (Jundong Yao). Standard two−dimensional grayscale wrist ultrasound images meeting the inclusion criteria were identified for each patient using Radiant DICOM Viewer 2021.1 (Medixant, Poznań, Poland) and converted to the Neuroimaging Informatics Technology Initiative (NIfTI) format (**Figure 2A**). A rectangular region of interest (ROI) covering the lesion area was then manually delineated on each image with ITK−SNAP software (www.itksnap.org) (**Figure 2B**). Pixel intensities within each ROI were normalized to a standard normal distribution N(0,1) using Z−score normalization.

To assess segmentation reproducibility, the same sonographer repeated the ROI delineation on 30 randomly selected cases one month after the initial segmentation. Reproducibility was quantified using the Dice similarity coefficient. The NIfTI images and corresponding ROI masks were subsequently imported into PyRadiomics (version 2.1.1) for high−throughput radiomics feature extraction.

In the training cohort, independent samples t−test was first used to select image features with significant differences (*P* < 0.05) in order to eliminate irrelevant feature parameters. Pearson correlation coefficients were calculated among radiomic features, and for any pair of features with *r* > 0.9, one was randomly retained. Finally, the least absolute shrinkage and selection operator (LASSO) regression algorithm was applied to identify the optimally sorted features, and features with non−zero coefficients were selected to derive the optimal subset of predictive features for assessing RA disease activity (**Figures 2E, F**). The features with non−zero coefficients were aggregated into a formula (**Appendix I**) to calculate the final radiomics score. The radiomics model was built using the ExtraTrees algorithm.

### 2.5 Construction of the combined model

In the training cohort, the MSK US model and the radiomics model were integrated using multivariate logistic regression to construct a combined predictive model. A nomogram was then developed to provide a graphical representation of this final model.

### 2.6 Development and validation of the nomogram

The nomogram was internally validated using the bootstrap method with 1,000 resamples. Its discriminatory performance was quantified by calculating the AUC. Calibration was assessed both graphically, via calibration plots, and statistically, using the Hosmer-Lemeshow goodness-of-fit test. The overall predictive accuracy was evaluated with the Brier score. Clinical utility was estimated through decision curve analysis (DCA)by calculating the net benefit across a range of threshold probabilities.

### 2.7 Statistical analysis

All statistical analyses were conducted using SPSS (version 27.0), MedCalc, and R statistical software. Categorical variables are presented as frequencies and percentages. Continuous variables following a normal distribution are expressed as mean ± standard deviation (SD), whereas those with a non-normal distribution are summarized as median and interquartile range (IQR; 25th-75th percentiles).

For between-group comparisons, categorical variables were analyzed using the chi-square test or Fisher’s exact test, as appropriate. Normally distributed continuous variables were compared using the independent samples t-test, and non-normally distributed variables were compared using the Mann-Whitney U test.

The radiomics model was built with the ExtraTrees algorithm. The MSK US model and the combined model were developed via multivariable logistic regression. Model performance was evaluated using ROC analysis. The “rms” and “pec” R packages were used to generate the nomogram and calibration curves, respectively. Internal validation was performed via bootstrap resampling implemented with the “caret” package. DCA was carried out using the “rmda” and “ggDCA” packages. A two-sided *P*-value < 0.05 was considered statistically significant.

## 3 Results

### 3.1 Patient characteristics

A total of 203 patients with RA were enrolled in this study. A cohort of 142 patients from the First Affiliated Hospital, College of Clinical Medicine of Henan University of Science and Technology served as the training cohort, while 61 patients from Affiliated Hospital of Traditional Chinese Medicine, Xinjiang Medical University (Fourth Clinical Medical College, Xinjiang Medical University) constituted the independent external test cohort. In the training cohort, there were 91 patients in clinical remission and 51 patients with clinically active disease, whereas in the test cohort, there were 37 patients in remission and 24 with active disease. No statistically significant differences in baseline characteristics were observed between the training and test cohorts (**Table 1**).

**Table 1:** Baseline characteristics of patients in the training and test cohorts.

Characteristic	Training cohort (n = 142)	Test cohort (n =61)	*P*
Age>57 (year)	76 (53.5%)	29 (47.5%)	0.434
Gender			0.502
Male	39 (27.5%)	14 (23.0%)	
Female	103 (72.5%)	47 (77.0%)	
RF (>61U/ml)	71 (50.0%)	36 (59.0%)	0.238
ESR (>54mm/h)	67 (47.2%)	28 (45.9%)	0.867
CRP (>32.83mg/L)	40 (28.2%)	18 (29.5%)	0.846
Bone erosion			0.903
Grade I	113 (79.6%)	49 (80.3%)	
Grade II	29 (20.4%)	12 (19.7%)	
Joint effusion			0.544
Grade 0	95 (66.9%)	37 (60.7%)	
Grade I	43 (30.3%)	23 (37.7%)	
Grade II	4 (2.8%)	1 (1.6%)	
Synovial hyperplasia			0.647
Grade I	82 (57.7%)	36 (59.0%)	
Grade II	58 (40.8%)	25 (41.0%)	
Grade III	2 (1.4%)	0 (0%)	
PDUS synovial blood flow			0.729
Grade 0	14 (9.9%)	8 (13.1%)	
Grade I	84 (59.2%)	35 (57.4%)	
Grade II	42 (29.6%)	18 (29.5%)	
Grade III	2 (1.4%)	0 (0%)	
SMI synovial blood flow			0.676
Grade I	23 (16.2%)	13 (21.3%)	
Grade II	78 (54.9%)	32 (52.5%)	
Grade III	41 (28.9%)	16 (26.2%)	

RF, rheumatoid factor; ESR, erythrocyte sedimentation rate; CRP, C-reactive protein; PDUS, power Doppler ultrasound; SMI, superb microvascular imaging.

### 3.2 Development and performance of the MSK US model

**Figure 3:**
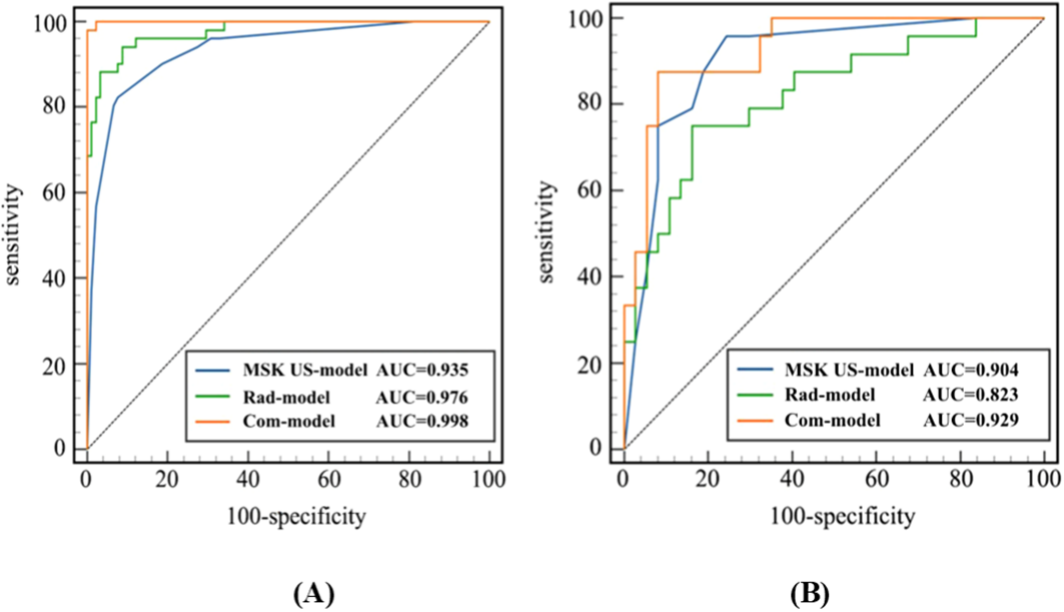
ROC curves of the three models in the training cohort **(A)** and the test cohort **(B)**. MSK US, musculoskeletal ultrasound; Rad−model, radiomics model; Com-model, combined model.

**Figure 4:**
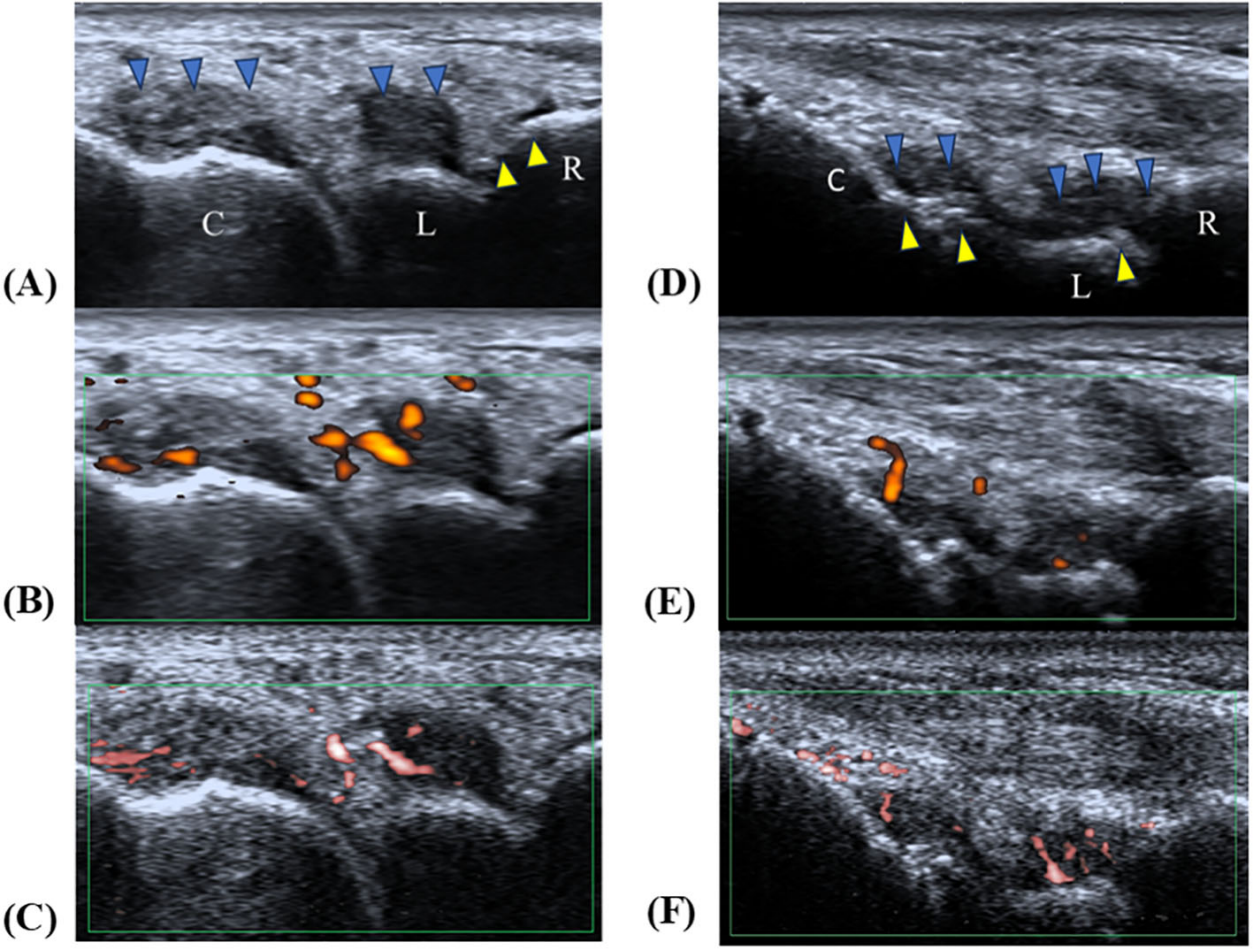
Case 1 **(A–C)**: representative images of moderately active synovitis. **(A)** A longitudinal grayscale ultrasound scan of the radiocarpal joint shows grade III synovial hyperplasia (blue arrow) and grade I bone erosion (yellow arrow). **(B)** PDUS in the same plane reveals grade II blood−flow signals, indicating active inflammation. **(C)** SMI in the same plane also demonstrates grade II blood−flow signals, confirming active synovial neovascularization. Case 2 **(D–F)**: Subclinical Synovitis in an RA Patient during Clinical Remission. **(D)** Longitudinal grayscale ultrasound of the radiocarpal joint demonstrates grade II synovial hyperplasia (blue arrow) and grade II bone erosion (yellow arrow). **(E–F)** In the same plane, PDUS shows grade I blood−flow signals, whereas SMI detects grade II signals. Both techniques confirm the presence of persistent subclinical synovial inflammation in this patient, with SMI exhibiting higher sensitivity than PDUS. R, radius; L, lunate; C, capitate; blue arrow, synovial hyperplasia; yellow arrow, bone erosion.

Univariate logistic regression analysis identified age > 57 years, rheumatoid factor (RF) > 61 U/mL, ESR> 54 mm/h, CRP > 32.83 mg/L, joint effusion grade I, joint effusion grade II, synovial hyperplasia grade II, PDUS synovial vascularity grade II, and SMI synovial vascularity grade III as predictors (**Table 2**). Multivariate logistic regression analysis was used to construct the MSK US model, and ESR > 54 mm/h, CRP > 32.83 mg/L, and SMI synovial blood flow grade III were identified as independent predictors of clinically active RA (**Table 2**). The MSK US model was constructed based on these independent predictors. In the training cohort, the AUC of the MSK US model was 0.935 (95% CI: 0.893–0.978), with a sensitivity of 82.4% and a specificity of 92.3%; in the test cohort, the AUC was 0.904 (95% CI: 0.825–0.983), with a sensitivity of 95.8% and a specificity of 75.7% (**Figure 3**; **Table 3**). **Figure 4** illustrates wrist synovitis ultrasound findings in two representative rheumatoid arthritis patients.

**Table 2:** Findings from both univariate and multivariate logistic regression analyses performed in the training cohort.

Characteristic	Univariate analysis		Multivariate analysis	
	95% CI	*P*	95% CI	*P*
Age>57 (year)	1.297-5.488	0.008		
Sex	0.992-4.480	0.053		
RF (>61U/ml)	1.111-4.537	0.024		
ESR (>54mm/h)	13.407-129.778	<0.001	5.057-77.943	<0.001
CRP (>32.83mg/L)	6.955-43.901	<0.001	1.309-15.399	0.017
Grade II bone erosion	0.393-2.176	0.857		
Joint effusion				
Grade 0	Reference			
Grade I	2.711-13.045	<0.001		
Grade II	1.045-106.978	0.046		
Synovial hyperplasia				
Grade I	Reference			
Grade II	2.562-11.572	<0.001		
Grade III	0	0.999		
PDUS synovial blood flow				
Grade 0	Reference			
Grade I	0.403-27.002	0.266		
Grade II	4.280-313.612	0.001		
Grade III	0	0.999		
SMI synovial blood flow				
Grade I	Reference			
Grade II	0.673-14.739	0.145	0.374-15.025	0.359
Grade III	6.467-163.835	<0.001	3.313-189.524	0.002

RF, rheumatoid factor; ESR, erythrocyte sedimentation rate; CRP, C-reactive protein; PDUS, power Doppler ultrasound; SMI, superb microvascular imaging.

**Table 3:** The diagnostic performance of the three models in both the training and testing cohorts.

Metric	MSK US-model		Rad-model		Com-model	
	Training cohort	test cohort	Training cohort	test cohort	Training cohort	Test cohort
AUC	0.935	0.904	0.976	0.823	0.998	0.929
95% CI	0.893-0.978	0.825-0.983	0.955-0.997	0.714-0.933	0.998-1.000	0.866-0.992
Sensitivity (%)	82.4	95.8	94.1	75.0	98	87.5
Specificity (%)	92.3	75.7	91.2	83.8	100	91.9

MSK US, musculoskeletal ultrasound; Rad−model, radiomics model; Com-model, combined model; AUC, area under the curve; CI, confidence interval.

### 3.3 Development and performance of the radiomics model

**Figure 5:**
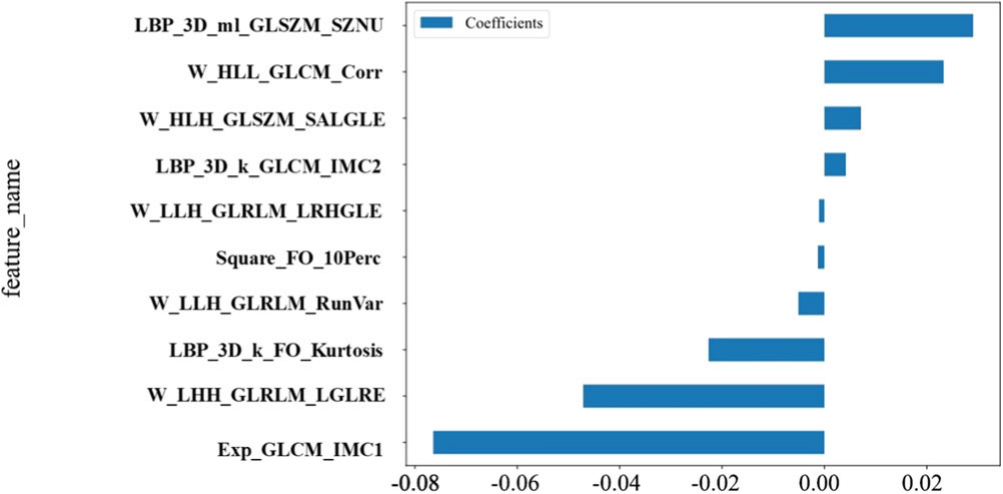
LASSO regression selected 10 ultrasound radiomics features and their corresponding coefficients plot. LBP, local binary pattern; W, wavelet transform; Exp, exponential filter; GLCM, gray-level co-occurrence matrix; GLSZM, gray-level size zone matrix; GLRLM, gray-level run-length matrix; Imc, informational measure of correlation; Corr, correlation; SALGLE, small area low gray level emphasis; LRHGLE, long run high gray level emphasis.FO, first-order; SZNU, size zone non-uniformity; RunVar, run variance; LGLRE, low gray level run emphasis.

A total of 1,561 features were extracted. These comprised shape features (n=14), first-order statistical features (n=306), and texture features derived from the gray-level co-occurrence matrix (GLCM, n=375), gray-level dependence matrix (GLDM, n=238), gray-level run-length matrix (GLRLM, n=272), gray-level size zone matrix (GLSZM, n=272), and the neighboring gray tone difference matrix (NGTDM, n=84). Ten radiomic features were identified through LASSO regression (**Figure 5**), and radiomics scores were calculated (**Appendix I**). The radiomics model was constructed using the ExtraTrees algorithm. In the training cohort, the AUC of the radiomics model was 0.976 (95% CI: 0.955–0.997), with a sensitivity of 94.1% and a specificity of 91.2%; in the test cohort, the AUC was 0.823 (95% CI: 0.714–0.933), with a sensitivity of 75.0% and a specificity of 83.8% (**Figure 3**; **Table 3**).

### 3.4 Development and performance of the combined model

A combined model integrating the MSK US model and the radiomics model was constructed. In the training cohort, the AUC of the combined model was 0.998 (95% CI: 0.998–1.000), with a sensitivity of 98.0% and a specificity of 100%; in the test cohort, the AUC was 0.929 (95% CI: 0.866–0.992), with a sensitivity of 87.5% and a specificity of 91.9% (**Figure 3**; **Table 3**). Internal validation was performed using the bootstrap method with 1,000 resampling iterations, yielding a mean AUC of 0.998.

### 3.5 Validation and clinical application of the nomogram

**Figure 6:**
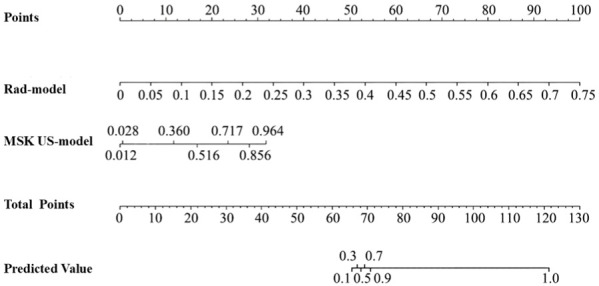
Nomogram for predicting RA disease activity MSK US, musculoskeletal ultrasound; Rad−model, radiomics mode.

**Figure 7:**
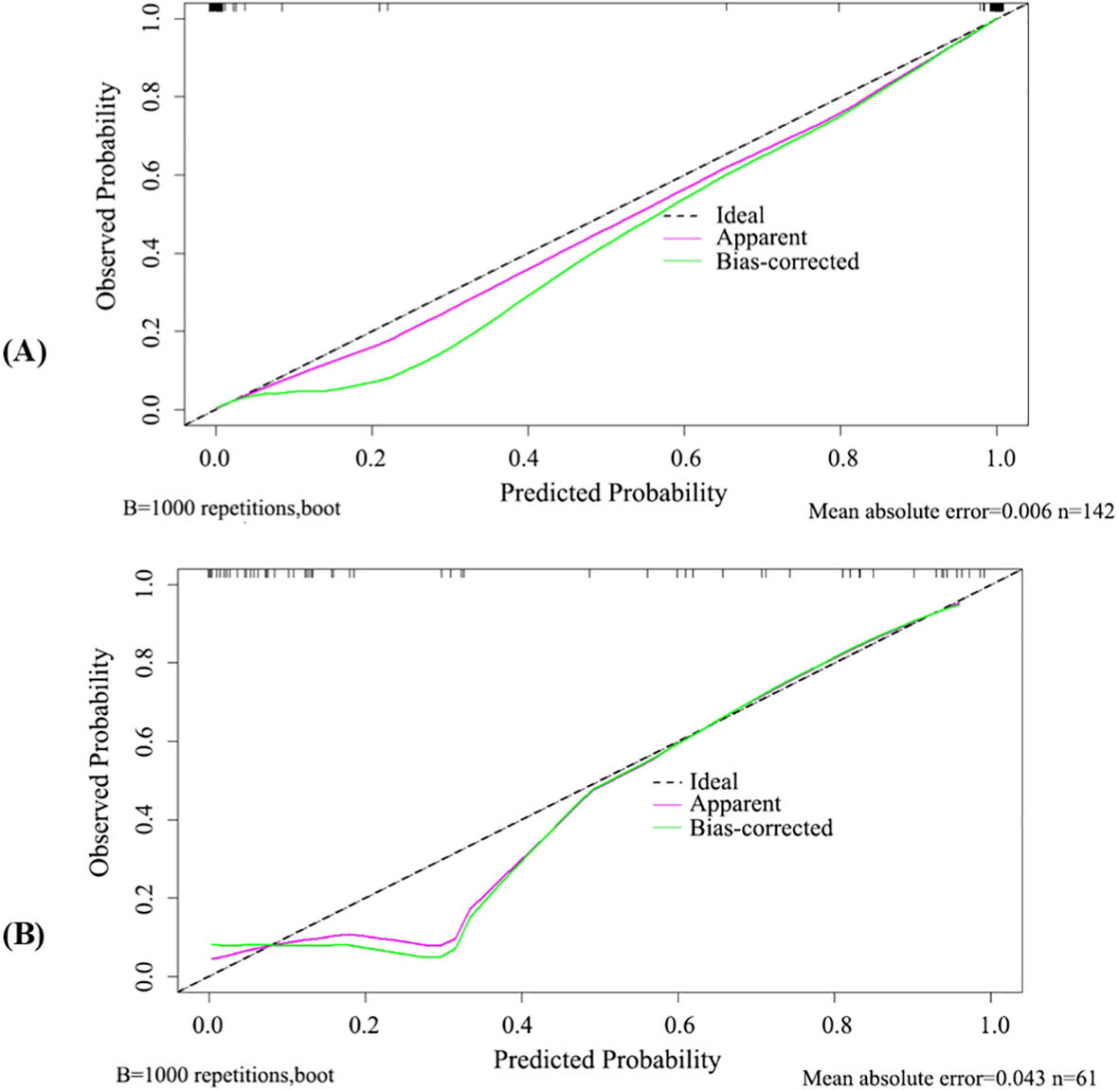
Calibration curves of the combined model in the training cohort **(A)** and test cohort **(B)**.

**Figure 8:**
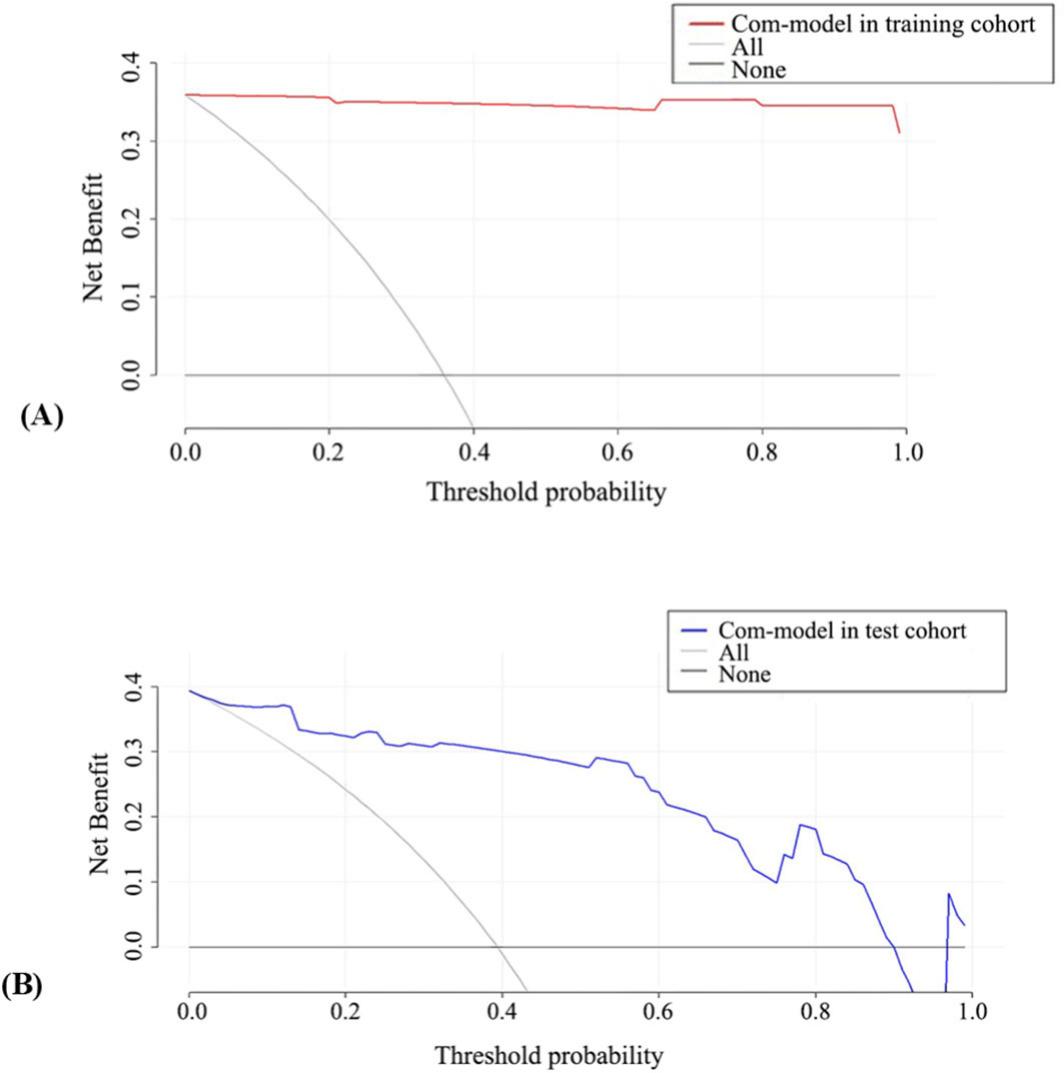
DCA of the combined model in the training cohort **(A)** and test cohort **(B)**. Com-model, combined model.

A nomogram was constructed based on the MSK US model and the radiomics model. Each model was assigned a corresponding score, and the sum of the scores was calculated. The probability corresponding to the total score represented the risk level of a patient being in the clinically active phase of RA (**Figure 6**). Calibration curve assessment demonstrated good agreement between the observed risk levels and the predicted risk probabilities (**Figure 7**). The Hosmer−Lemeshow goodness−of−fit test indicated that the combined model showed good calibration in the training cohort (P = 0.998), whereas its calibration in the test cohort was significantly inferior (P < 0.001). The Brier scores for the training and test cohorts were 0.01 and 0.10, respectively, indicating good overall performance of our predictive model. Decision curve analysis showed that the model provided significant net benefit across threshold probability ranges of 0.02–0.80 in the training cohort and 0.12–0.78 in the test cohort (**Figure 8**).

## 4 Discussion

In this study, we successfully developed and validated a combined model integrating conventional MSK US indicators with ultrasound radiomics features for assessing disease activity in RA. The experimental data demonstrated that the model achieved an AUC of 0.998 in the training cohort and 0.929 in the test cohort, and exhibited significantly superior diagnostic performance compared with either the MSK US model or the radiomics model alone. This provides a novel tool for the objective and accurate assessment of disease activity in RA.

Univariate logistic regression analysis showed that ESR > 54 mm/h, CRP > 32.83 mg/L, and RF > 61 U/mL were each significantly associated with clinically active RA. Further multivariate logistic regression analysis identified ESR > 54 mm/h and CRP > 32.83 mg/L as independent predictors of active RA, whereas RF did not enter the final model. As acute-phase reactants, the predictive value of ESR and CRP stems from the cytokine-driven hepatic synthesis of CRP and alterations in plasma protein composition during the inflammatory process in RA, which in turn accelerate ESR. Both markers dynamically reflect systemic inflammatory burden and provide systemic, real-time indicators that are highly synchronized with local synovial inflammation. This systemic perspective complements the local joint inflammation captured by SMI synovial vascularity signals, forming a “systemic-local” synergy that enables the model to capture the multidimensional information of disease activity more comprehensively and robustly, serving as a bridge connecting microscopic molecular events with macroscopic clinical assessments (18, 19). In contrast, although RF holds significant value in the diagnosis and long-term prognosis of RA, it did not emerge as an independent predictor in the present model. This suggests that different biomarkers provide information at varying levels of hierarchy and temporal relevance in different clinical contexts: RF tends to reflect underlying immune abnormalities and chronic background, whereas in the assessment of real-time inflammatory activity, its incremental information is already covered by dynamic indicators such as ESR and CRP, as well as by inflammatory angiogenesis directly visualized by SMI (20). Therefore, when constructing predictive models aimed at real-time disease activity, priority should be given to integrating dynamic indicators that change synchronously with the inflammatory process, while reserving relatively stable markers such as RF for disease classification or long-term prognostic assessment, where they may be of greater value.

Second, SMI synovial blood flow grade III was established as a key independent imaging predictor. This finding has a clear pathophysiological basis: synovial microvascular proliferation is a central component of inflammatory activity in RA. During the active phase of RA, pro-inflammatory cytokines drive marked dilation and proliferation of capillaries in the sub-synovial layer, resulting in the formation of an extensive neovascular network. SMI is capable of sensitively detecting the very low-velocity microvascular flow signals within this network, representing a core technical advantage over conventional PDUS. Therefore, SMI synovial blood flow grade III is not merely an imaging finding but also a macroscopic reflection of active angiogenesis and inflammatory cascade reactions within the synovial tissue, serving as a typical imaging marker of diffuse vascularization in highly active synovitis (21).

In our model, SMI synovial blood flow grade III emerged as an independent predictor, whereas none of the PDUS grades entered the multivariate model. Although PDUS synovial blood flow grade II was significant in univariate analysis, it was eliminated in the multivariate analysis; PDUS synovial blood flow grades I and III were not significant even in univariate analysis. These findings suggest that PDUS grading has limited overall discriminative ability for determining the active phase of RA, whereas SMI, by detecting low-velocity microvascular flow, more accurately reflects the true inflammatory burden of the synovium (22). Thus, SMI synovial blood flow grade III serves as a key imaging threshold for distinguishing active RA from remission. Based on the results of this study, we propose a tiered ultrasound application strategy in clinical practice: PDUS should be used as a first-line screening and routine follow-up tool, given its well-established grading system and relatively standardized operation, which are sufficient to support most routine clinical decisions (23); when faced with patients in clinical remission who require differentiation of subclinical synovitis, when PDUS findings are inconsistent with clinical presentation, or when assessment of very early treatment response is needed, SMI should be employed as an advanced imaging tool. The higher sensitivity of SMI for detecting microvascular flow provides complementary information, thereby improving diagnostic accuracy and guiding individualized treatment (24).

Several limitations of this study warrant consideration. First, this was an exploratory study without a precalculated sample size. Although the total of 203 patients falls within the typical range for radiomics studies, the sample size remains relatively limited, and future studies should conduct formal sample size estimation based on the effect sizes observed here. Second, external validation was performed using an independent center [Affiliated Hospital of Traditional Chinese Medicine, Xinjiang Medical University (Fourth Clinical Medical College, Xinjiang Medical University)], providing rigorous evidence for model generalizability; however, the scope of validation remains narrow. Future studies should include multicenter, multivendor data to further assess cross−center stability. Finally, radiomic feature extraction relied on manual segmentation of regions of interest—a common challenge in this field. Future work could integrate deep learning−based automatic segmentation techniques to reduce manual dependency and improve reproducibility.

The methodological framework developed in this study demonstrates clear potential for extension, with a primary direction lying in vertical translation—applying the model to predict individual patient responses to specific therapeutic regimens (e.g., biologic agents) to advance from diagnostic assessment toward treatment prediction—and a secondary direction involving horizontal expansion into another significant application of MSK US in rheumatology: the evaluation of enthesitis, defined as inflammation at the sites where tendons, ligaments, or joint capsules insert into bone. As a core pathological feature of conditions such as spondyloarthritis, enthesitis is considered a fundamental lesion in rheumatic diseases, and high-resolution ultrasound effectively visualizes key entheseal abnormalities, including Doppler flow signals, tendon thickening, cortical bone irregularities (erosions or depressions), and enthesophyte formation. For example, Ricci V et al. systematically correlated high-frequency ultrasound findings with microanatomy to delineate characteristic inflammatory changes in the subcutaneous tissue, tendons, and pulleys of patients with dactylitis, illustrating the utility of high-resolution ultrasound in characterizing such fundamental lesions (25); furthermore, a multicenter study by Di Matteo A et al. confirmed that the detection of PDUS flow signals and bone erosions at the enthesis are among the most discriminative sonographic features for spondyloarthritis, and their work established specific ultrasound criteria for active enthesitis (entheseal thickening with a hypoechoic area plus PD flow of grade ≥1), which identified active enthesitis in 89.9% of SpA patients and helped differentiate SpA from non-inflammatory entheseal pathologies such as osteoarthritis and fibromyalgia (26). In current practice, however, the recognition and integration of these imaging features remain largely dependent on operator experience and subjective semi-quantitative scoring, limiting standardization, reproducibility, and cross-center comparability. The combined model validated here provides a methodological blueprint for addressing this limitation, as future studies could apply an analogous analytical pipeline to entheseal ultrasound images; by quantifying textural features, blood-flow distribution patterns, and cortical morphology, such work may yield objective tools for diagnosing enthesitis, distinguishing inflammatory from degenerative changes, and monitoring therapeutic response, which would extend the quantitative capabilities of MSK US from the joint space to periarticular structures and enable a more holistic assessment of musculoskeletal disease.

## 5 Conclusion

In conclusion, this study developed and validated a combined predictive model integrating musculoskeletal ultrasound and radiomics for assessing disease activity in rheumatoid arthritis. The model achieved an AUC of 0.929 in independent external validation, demonstrating significantly superior diagnostic performance compared with either single-modality model. Its clinical value lies in providing clinicians with an objective and accurate assessment tool, facilitating the optimization of individualized treatment strategies and supporting the achievement of treat-to-target goals in patients with RA.

## Data Availability

The original contributions presented in the study are included in the article/**Supplementary Material**. Further inquiries can be directed to the corresponding author.
