# Supplementary material for: Development and validation of a combined ultrasound−radiomics model for assessing rheumatoid arthritis disease activity: a prospective, two−center diagnostic study
Source: Front Immunol. 2026 Apr 27;17:1669766. doi: 10.3389/fimmu.2026.1669766 (PMC13158220; doi:10.3389/fimmu.2026.1669766)
Supplement: Supplementary file 1 [file Table1.docx]

**Appendix I**

label = 0.3694581280788216 -0.076422 * exponential_glcm_Imc1 -0.022623 * lbp_3D_k_firstorder_Kurtosis +0.004189 * lbp_3D_k_glcm_Imc2 +0.029044 * lbp_3D_m1_glszm_SizeZoneNonUniformity -0.001274 * square_firstorder_10Percentile +0.007133 * wavelet_HLH_glszm_SmallAreaLowGrayLevelEmphasis +0.023286 * wavelet_HLL_glcm_Correlation -0.047112 * wavelet_LHH_glrlm_LowGrayLevelRunEmphasis -0.001056 * wavelet_LLH_glrlm_LongRunHighGrayLevelEmphasis -0.005091 * wavelet_LLH_glrlm_RunVariance
